# Fluorine Transformation Along the Chain from Phosphate Rock to Phosphogypsum and Phosphogypsum-Based Cemented Backfill

**DOI:** 10.3390/ma19173773

**Published:** 2026-09-04

**Authors:** Yanan Zhou, Ying Shi, Chendi Min, Quanqi Zhu

**Affiliations:** 1 School of Resources, Environment and Safety Engineering, Hunan University of Science and Technology, Xiangtan 411201, China; yanan.zhou@hnust.edu.cn (Y.Z.); mincd@hnust.edu.cn (C.M.); 2 School of Resources and Safety Engineering, Central South University, Changsha 410083, China; shiyingfriend@csu.edu.cn

**Keywords:** fluorine, phosphogypsum, cemented paste backfill, speciation transformation, leaching behavior

## Abstract

Fluorine in phosphogypsum (PG) poses a persistent leaching risk, and PG-based cemented paste backfill (CPB) offers a practical option for in situ stabilization. In this study, fluorine transformation from phosphate rock (PR) to PG and further to CPB was quantified using acid dissolution for total fluorine (TF) determination and sequential extraction for fluorine speciation. Pearson correlation analysis was used to examine statistical relationships among fluorine fractions. TF decreased progressively during wet-process phosphoric acid production, PG stockpiling, and CPB preparation. However, the proportion of mobile fluorine increased from PR to CPB, indicating incomplete reversal of process-induced fluorine activation. CPB immobilized most mobile fractions, increasing the residual fraction to 88.83–97.17% of TF and achieving fluorine transfer efficiencies of 93.59–94.75%. Speciation results showed marked decreases in water-soluble fluorine (Ws-F) and exchangeable fluorine (Ex-F) in CPB, while Pearson correlation analysis further suggested strong statistical associations among Ws-F, Ex-F, and other fractions, suggesting potential interconversion pathways under alkaline, Ca-rich CPB conditions. Nevertheless, a Ws-F-dominated mobile fraction remained in CPB, accounting for 2.83–11.19% of TF, and governed the long-term leaching risk. Therefore, assessing CPB based on process-oriented fluorine speciation, rather than merely on TF content, is critical for optimizing PG-based CPB formulations and achieving the safe reutilization of PG.

## 1. Introduction

Fluorine is an essential trace element that is ubiquitously distributed among various environmental matrices, including air, soil, and groundwater, and it exerts both beneficial and adverse physiological effects [1,2,3,4]. While appropriate concentrations of fluorine support human health, excessive exposure has been demonstrated to induce pathophysiological outcomes, such as dental fluorosis, skeletal fluorosis, and thyroid dysfunction. Thus, this results in a biphasic physiological response [5,6]. The presence of fluorine in the environment is a result of both natural and anthropogenic sources. Natural processes, including volcanic activity, marine aerosol deposition, and mineral weathering, constitute the primary sources of baseline environmental fluorine. In contrast, anthropogenic activities such as coal-fired power generation, brick manufacturing, and phosphate chemical processing have significantly increased environmental fluorine concentrations over recent decades [7,8,9]. Elevated fluorine concentrations attributed to anthropogenic emissions have become a critical environmental issue [10].

Rapid expansion of industrial activities has intensified the demand for phosphorus products, positioning the phosphorus industry as a dominant anthropogenic source of fluoride emissions [11,12,13]. Phosphate rock (PR), composed mainly of fluorapatite (Ca_5_(PO_4_)_3_F), serves as the principal raw material for the production of phosphate chemicals via an acidulation process. The reaction can be represented by the following Equation (1):

(1) $$Ca_{5}{\left(PO_{4}\right)}_{3}F+5H_{2}SO_{4}+10H_{2}O\to 5CaSO_{4}\cdot nH_{2}O+3H_{3}PO_{4}+HF$$

During this process, approximately 10–15% of the fluorine is released as gaseous species, while the remaining 85–90% is retained in the by-product phosphogypsum (PG) in a variety of chemical species [14,15,16]. The resulting PG is mainly composed of CaSO_4_·2H_2_O but also contains various impurities inherited from the PR and introduced during the production process, including phosphates, fluorides, and naturally occurring radionuclides such as ^226^Ra [17]. Among these impurities, fluorine poses a particularly acute environmental concern due to its high mobility and toxicity. Due to its high permeability, PG facilitates the leaching of soluble fluorides upon exposure to rainwater, with concentrations reported to reach up to 4.25 mg/L [18,19,20]. Additionally, stored PG is known to release volatile fluoride compounds, such as SiF_4_ and HF, leading to fluoride accumulation in nearby soils, where concentrations may reach 4667 mg/kg, significantly above natural background levels [21,22]. Currently, more than 52 countries are estimated to maintain substantial PG stockpiles, exceeding six billion tonnes in total, with annual increases of approximately 200–300 million tonnes [23,24,25]. Thus, controlling fluoride release from PG is a pressing global environmental challenge.

The cemented backfill technique has emerged as an effective method for solidification and stabilization of impurities in solid wastes [26,27]. Recent studies have highlighted the potential of using PG as an aggregate in in situ PG-based cemented backfill (CPB), supporting sustainable mining activities by reducing fluoride pollution [28,29]. Interactions between fluorine in PG and hydration products from cementitious materials have been shown to significantly decrease the concentrations of soluble fluoride leached from the backfill [30,31]. For instance, Li et al. [32] found that incorporating PG with an initial soluble fluoride concentration of 1355 mg/L into the backfill reduced the corresponding slurry concentration to 257–615 mg/L. Similarly, Liu et al. [33] observed a reduction in soluble fluoride concentration from 113.72 mg/L in raw PG to 11.95 mg/L in the PG-based CPB leachate.

Despite significant progress in understanding fluoride behavior in PG and cemented backfill, several knowledge gaps remain. Previous studies have primarily classified fluoride in PG into soluble and insoluble fractions, providing valuable insights into overall mobility trends [34]. However, this simplified classification may not fully capture the complexity of fluorine speciation. In reality, fluoride occurs in many species, including water-soluble fluorine (Ws-F), exchangeable fluorine (Ex-F), iron-manganese oxide fluorine (Fe/Mn-F), organically bound fluorine (Or-F), residue fluorine (Res-F), etc., each with distinct environmental behaviors [35,36]. Factors such as temperature, moisture, and especially changes in storage conditions can induce transformations among different fluoride species, affecting their solubility and mobility in the environment [37,38]. Moreover, pH has a critical influence on fluoride speciation [39]. Within the context of cemented PG backfilling, PR, PG, and PG-based CPB represent the primary fluorine-bearing materials, each exhibiting distinct pH and stored conditions. Nevertheless, previous research has primarily concentrated on changes in the soluble fluoride of relevant materials, with relatively less attention devoted to the specific fluoride species.

Therefore, the objective of this study is to comprehensively assess the specific species and transformations of fluoride from PR to PG and further to the PG-based CPB. PR, as well as both fresh and aged PG samples, were collected from the phosphate mine. PG-based CPB samples were prepared under controlled laboratory conditions to simulate industrial practices. The total fluorine (TF) content was measured using an acid dissolution method, and the fluoride species were analyzed using a modified Tessier five-step sequential extraction procedure. The results from this study are expected to deliver a comprehensive assessment of the environmental risks associated with fluorine during the cemented backfilling process, thereby enabling the development of more effective mitigation strategies.

## 2. Materials and Methods

### 2.1. Materials

#### 2.1.1. Raw Materials

The PR and composite binder were sourced from a phosphorus mine in Guizhou, China. The composite binder, containing negligible fluoride content, was formulated with phosphorous slag, fly ash, cement clinker, and slaked lime at a mass ratio of 4:1:1:0.64–0.80 [40]. The fresh and aged PG samples were collected from an open stockpile site of a mine backfill plant. To preserve in situ characteristics, all samples were immediately sealed in clean plastic bags and kept under controlled conditions prior to testing.

**Figure 1:**
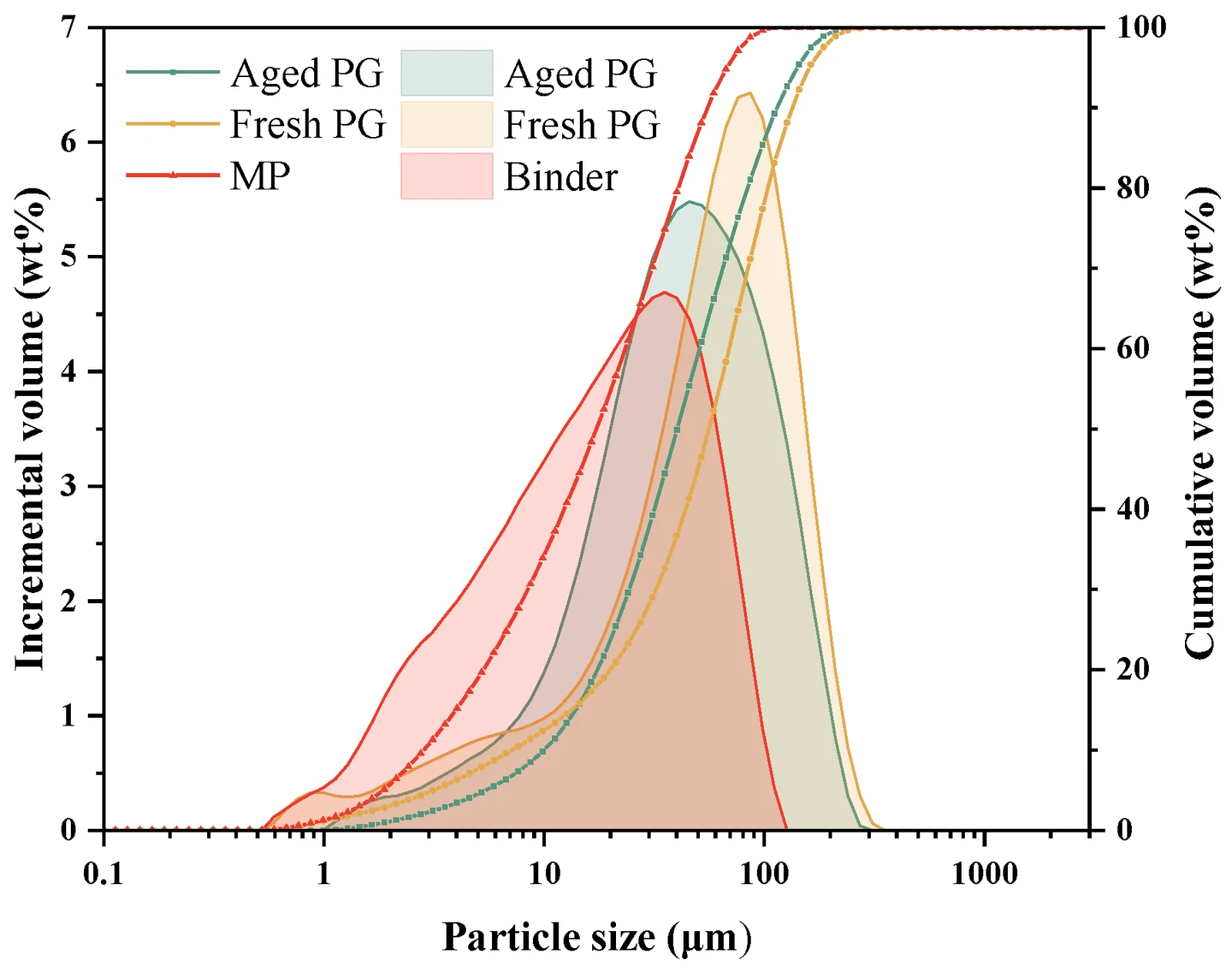
Particle size distribution of raw materials.

**Figure 2:**
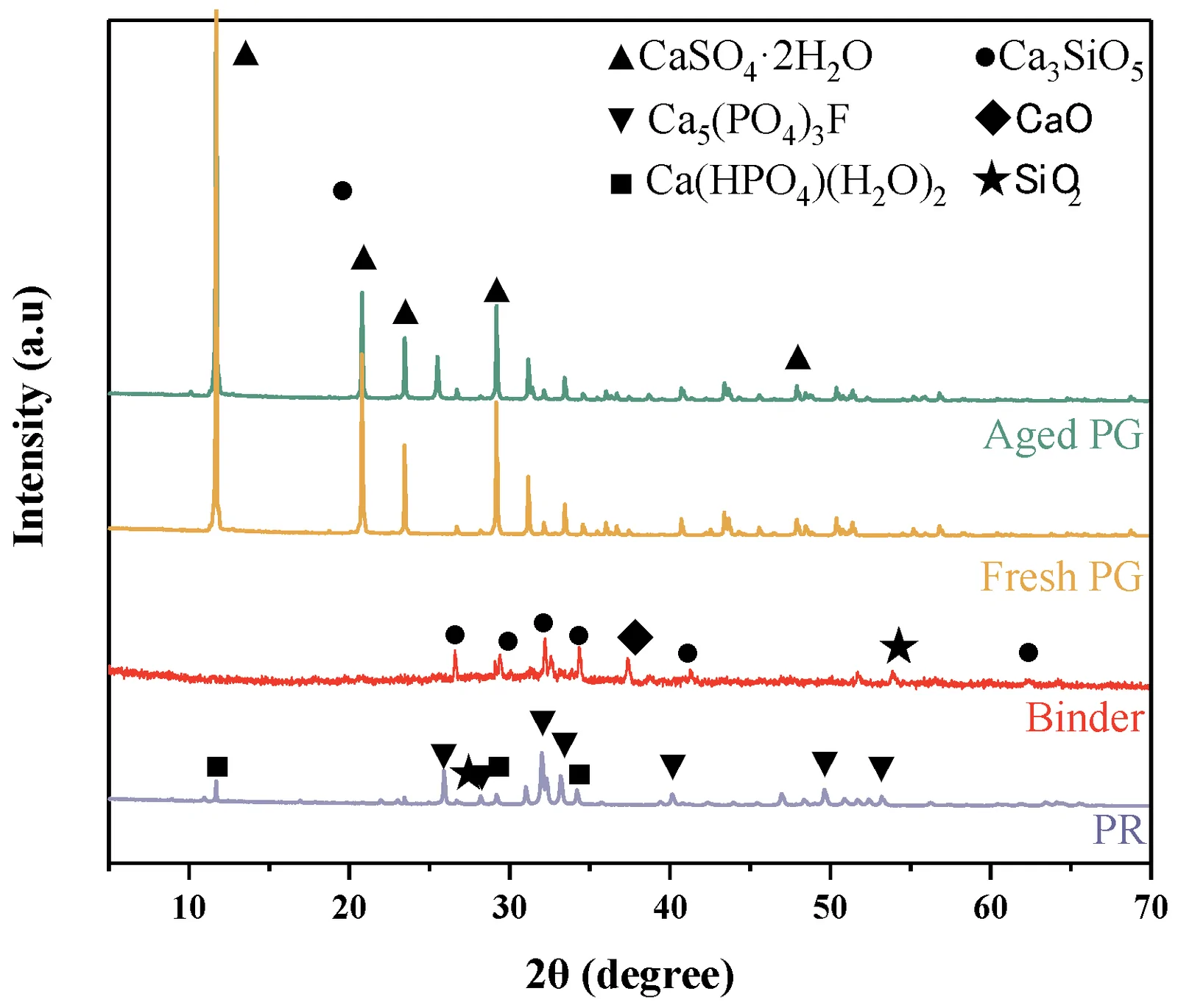
The XRD patterns of the raw materials.

The particle size distribution of the raw materials was determined using a particle size analyzer (Malvern Instruments, Malvern, UK). The mineral composition was characterized by X-ray diffraction (XRD, Bruker D8 Advance, Karlsruhe, Germany) over a 2θ range of 5–80° at a scan rate of 5°/min. Phase content calculation was conducted using the Rietveld method. The corresponding particle size distribution curves and XRD patterns are presented in Figure 1 and Figure 2, respectively, as baseline characterization of the raw materials.

#### 2.1.2. Sample Preparation

PG-based CPB samples were prepared at a mass ratio of 1:4:5 (composite binder: PG: deionised water), reflecting operational formulations commonly used in mining practice and enabling the planned speciation assessment [34,40]. The slurry was thoroughly mixed mechanically at 200 rpm for 30 min and then cast into 40 mm cubic molds. After bleeding ceased, the molds were transferred under 20 ± 2 °C and 90 ± 5% relative humidity. Specimens were demolded near final setting and cured for 28 days under identical conditions. Before subsequent testing, all samples were immersed in anhydrous ethanol to terminate hydration, ensuring comparability of fluoride speciation across materials.

### 2.2. Methods

#### 2.2.1. Total Fluorine Content in Phosphate Rock

**Figure 3:**
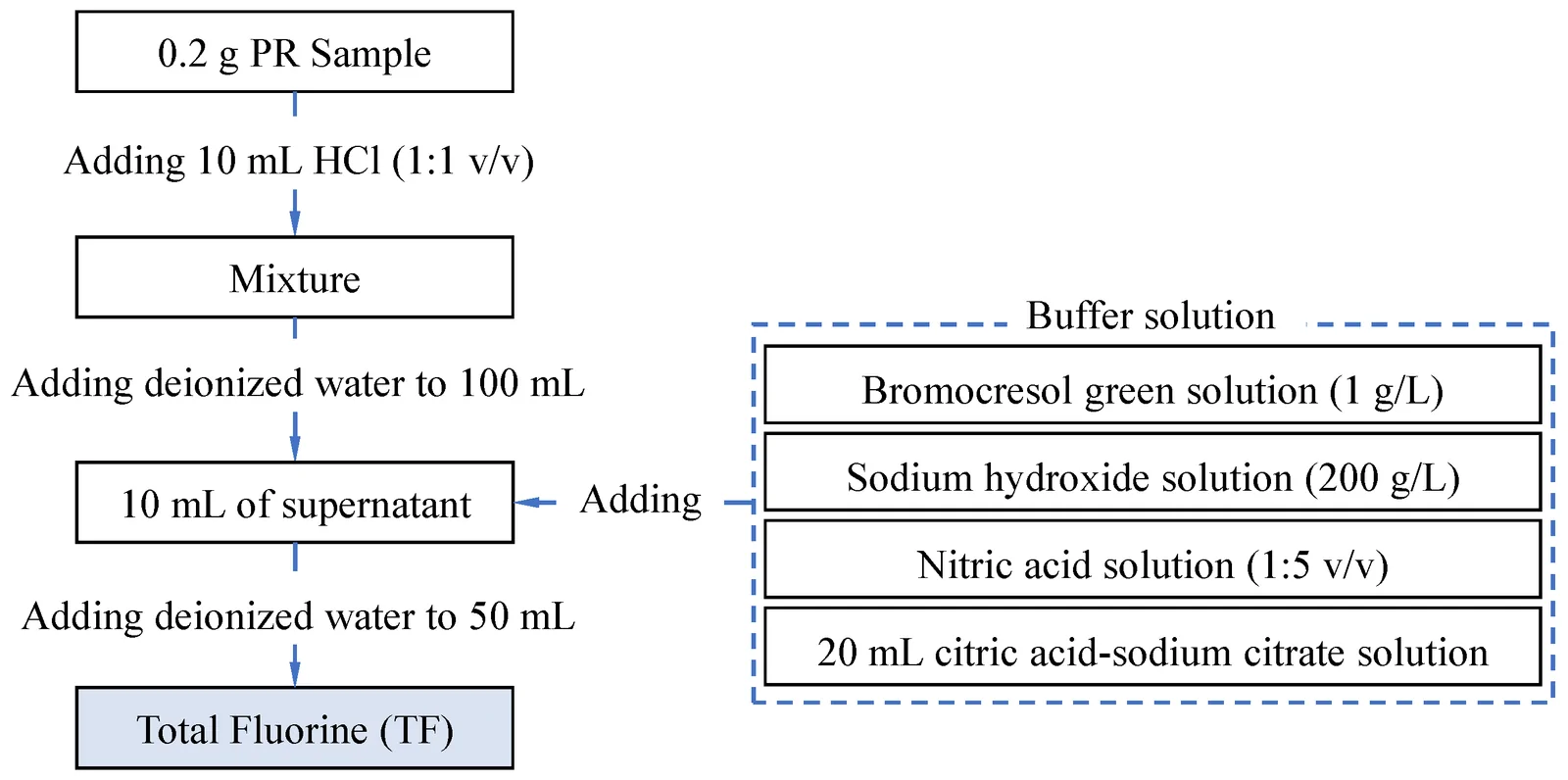
Procedure for determining the total fluorine content in PR.

The TF content in PR was determined according to the Chinese standard GB/T 1872–1995 [41]. First, PR samples were dried at 105–110 °C to constant mass and cooled to room temperature. Next, the dried samples were ground and passed through a 125 μm sieve to ensure uniform particle size. A 0.2 g aliquot of the sieved PR was thoroughly digested with 10 mL of HCl (1:1 *v*/*v*) in a 50 mL beaker and allowed to settle for 30 min. Following this, the mixture was diluted to 100 mL with deionized water. Then, a 10 mL aliquot of the supernatant was transferred to a new container, and a buffer solution was added (composition detailed in Figure 3). Finally, the supernatant volume was adjusted to 50 mL with deionized water. The fluoride concentration in the supernatant was determined in triplicate, and mean values were recorded. The experimental procedure is illustrated in Figure 3.

#### 2.2.2. Total Fluorine Content in PG and PG-Based Cemented Backfill

**Figure 4:**
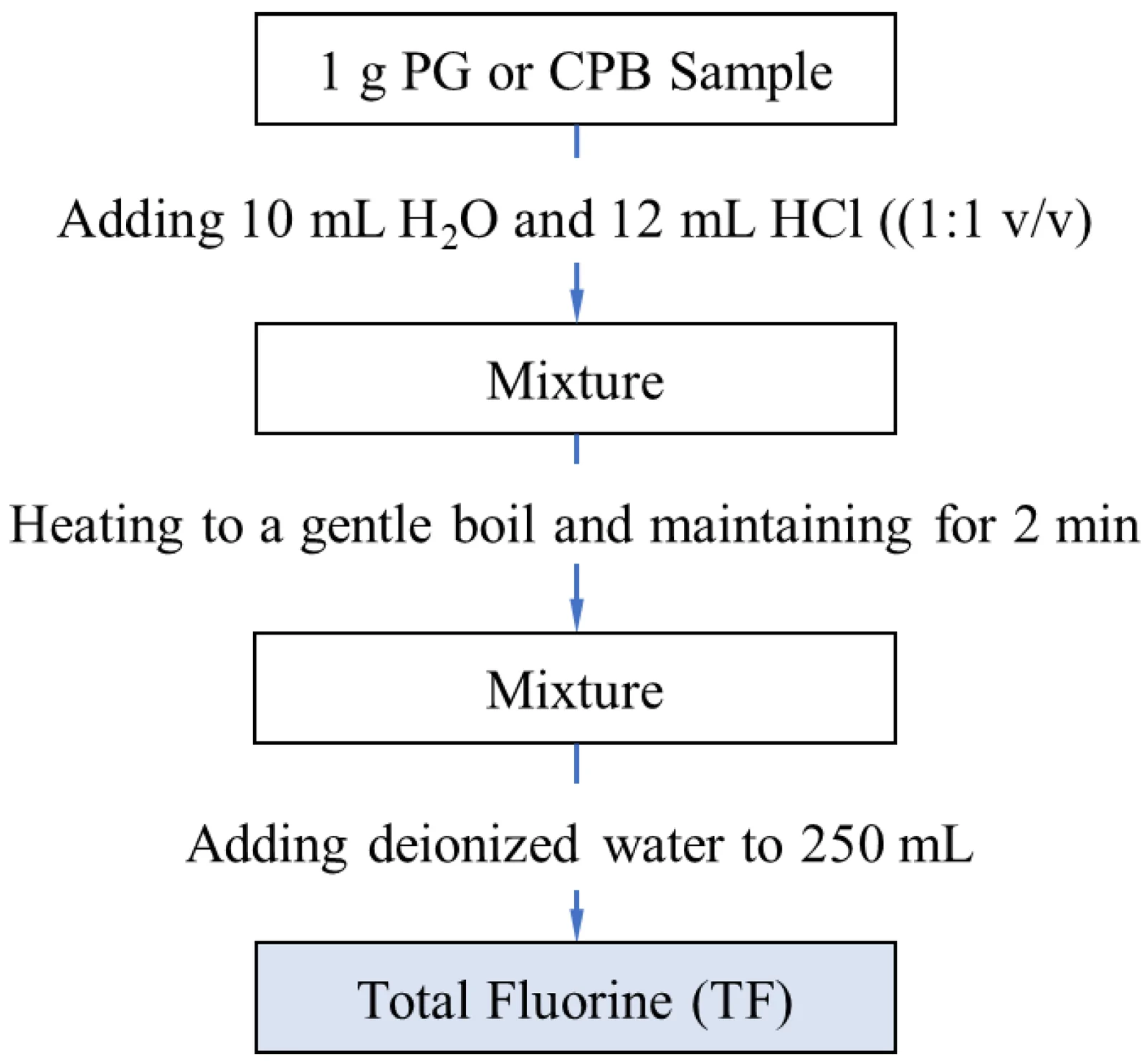
Procedure for determining the total fluorine content in PG and PG-based CPB.

The TF in PG and PG-based CPB was determined according to JC/T 2073-2011 [42]. First, the PG and backfill samples were dried at 40 ± 5 °C to constant mass and cooled to room temperature. The dried samples were then crushed and sieved through an 80 μm sieve. Next, 1 g of the sieved sample was thoroughly mixed with 10 mL of deionized water and 12 mL of HCl (1:1 *v*/*v*) in a 100 mL beaker. Subsequently, the mixture was brought to a gentle boil in a water bath and maintained for 2 min. Finally, the mixture was diluted to 250 mL with deionized water. The supernatant was analyzed in triplicate, and the average value was recorded. The complete experimental procedure is illustrated in Figure 4.

#### 2.2.3. Fluoride Species in Samples

Fluoride species in PR, PG, and PG-based CPB were analyzed using a modified Tessier five-step sequential extraction, as described previously for fluorine speciation in solid matrices [43,44,45]. The specific steps of the method are as follows:

Step 1: Water-soluble fluorine (Ws-F)

First, 5 g of crushed sample (particle size < 0.15 mm) was mixed with 50 mL of deionized water in a capped container. The mixture was shaken at 220 rpm for 30 min at 25 °C, allowed to settle for a few minutes, and then centrifuged at 4000 rpm for 10 min to collect the supernatant. Second, 10 mL of deionized water was added to the residue, followed by shaking and centrifugation again. This washing was repeated three times, combining all supernatants. The combined supernatants were evaporated in a water bath to a volume of less than 50 mL, then diluted to 50 mL with deionized water, and the fluoride concentration was measured.

Step 2: Exchangeable fluorine (Ex-F)

The Step 1 residue was treated with 50 mL of 1 mol/L MgCl_2_ solution. After shaking (1 h, 25 °C) and centrifugation, the residue was washed twice. Supernatants were processed as in Step 1.

Step 3: Iron-manganese oxide fluorine (Fe/Mn-F)

The Step 2 residue was reacted with 50 mL of 0.5 mol/L HONH_3_Cl. Following 1 h of shaking and centrifugation, the residue was washed twice. Supernatants were processed as in Step 1.

Step 4: Organically bound fluorine (Or-F)

The Step 3 residue was treated with 20 mL of acidified H_2_O_2_ (pH = 2 HNO_3_ *v*/*v*). The container was covered and stirred occasionally at room temperature for 2 h. The mixture was then evaporated to near-dryness (less than 5% residual moisture) in a 90 °C water bath to remove H_2_O_2_ completely. Subsequently, 50 mL of 3.2 mol/L NH_4_Ac was added. The mixture was shaken for 1 h and then centrifuged. Supernatants were processed as in Step 1.

Step 5: Residual fluorine (Res-F)

The residual fluorine was calculated as the difference between the TF and the sum of other species, as shown in Equation (2):

(2) Res-F=TF-Ws-F-Ex-F-Fe/Mn-F-Or-F

**Figure 5:**
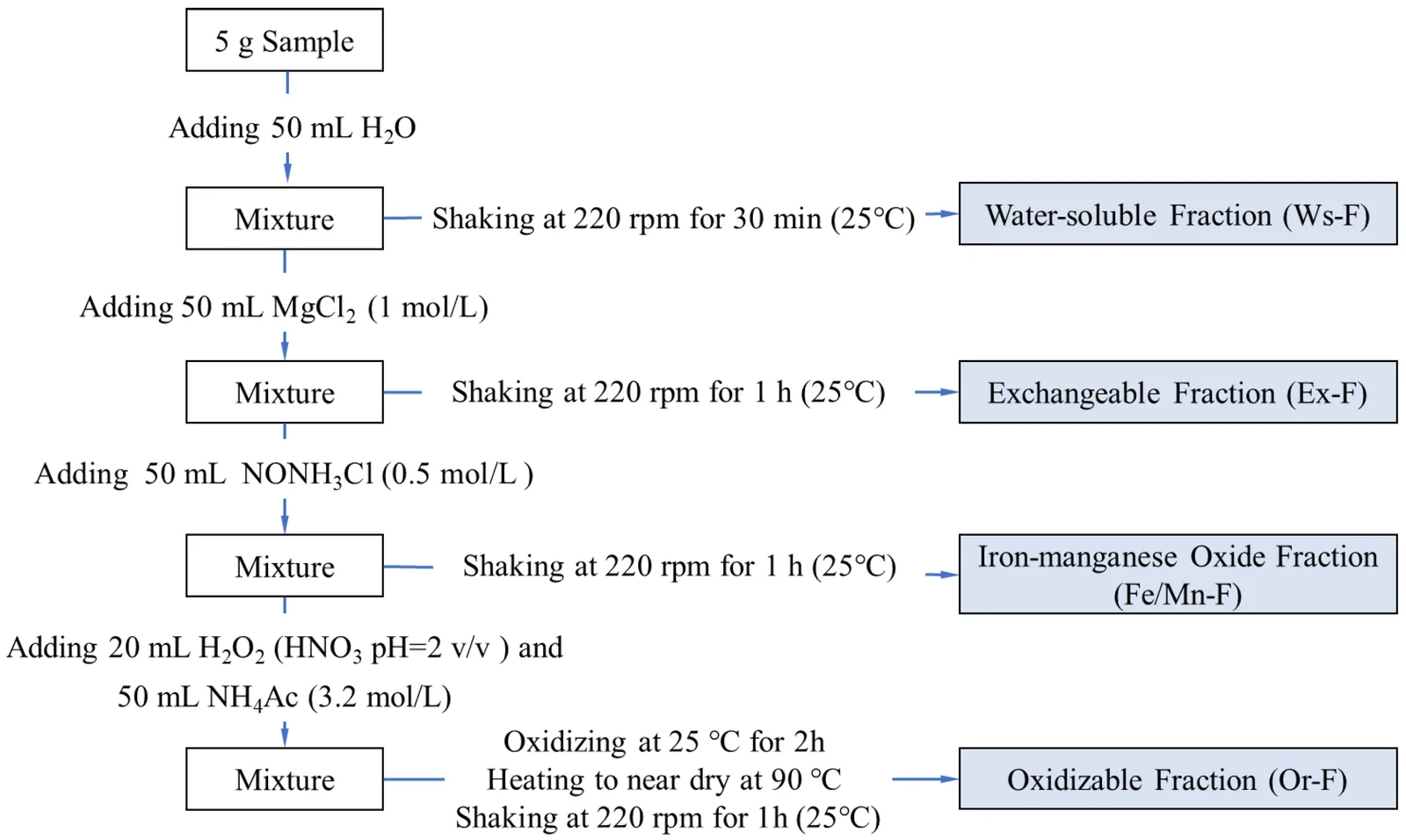
Procedure for determining fluoride species in PR and PG-based CPB.

The complete experimental procedure is illustrated in Figure 5.

#### 2.2.4. Total Fluorine Transfer Efficiency

To evaluate the transfer efficiency of TF from PG to backfill, a modified element transfer factor (ETF) was calculated using Equation (3), accounting for the binder-to-aggregate ratio, slurry concentration, and bleeding rate during the preparation of the PG-based CPB [46,47].

(3) $$ETF=\frac{1}{\alpha }\cdot \frac{m_{Backfill}}{\omega \cdot m_{PG}}\times 100$$

where *α* is the mass normalization coefficient, considering that 1 kg of the PG generates approximately 1.875 kg of the cemented backfill; *ω* is the ratio (about 0.75) of dry PG to the solid mass for making backfill; *m_Backfill_* and *m*_PG_ are the masses of cemented backfill and PG, respectively.

#### 2.2.5. Fluoride Mobility Assessment

Fluoride species were operationally categorized into mobile and immobile fractions based on environmental liability to assess the mobilization potential. Ws-F, Ex-F, Fe/Mn-F, and Or-F were defined as the mobile fraction, while the Res-F was defined as the immobile fraction [48]. The relative abundance of fluoride in the mobile and immobile fractions was calculated using Equations (4) and (5):

(4) $$\omega F_{M}=\frac{F_{M}}{F_{TF}}\times 100\%$$

(5) $$\omega F_{IM}=\frac{F_{IM}}{F_{TF}}\times 100\%$$

where *F_M_* is the content of mobile fluoride in the sample, which is the sum of Ws-F, Ex-F, Fe/Mn-F and Or-F; *F_IM_* is the content of immobile fluoride; *F_TF_* is the TF content.

#### 2.2.6. Chemical Analysis

Leachates obtained from the above tests were subjected to chemical analysis to measure fluoride concentrations. First, the leachates were filtered through a 0.45 µm filter to obtain a clear supernatant, ensuring suspended particles were removed. Then, the fluoride concentration in this filtered supernatant was measured using an ion meter (Mettler Toledo, Greifensee, Switzerland) equipped with a fluoride ion-selective electrode (Leici, Shanghai, China), with a calibration range of 0.1–100 mg/L (r^2^ = 0.9998).

Unless otherwise stated, all reagents used in the chemical analysis were of analytical grade or higher. Sodium fluoride (NaF, ≥99%), magnesium chloride hexahydrate (MgCl_2_·6H_2_O, ≥98%), sodium acetate (CH_3_COONa, ≥99%), hydroxylamine hydrochloride (NH_2_OH·HCl, ≥98.5%), hydrogen peroxide (H_2_O_2_, 30%), ammonium acetate (CH_3_COONH_4_, ≥98%), and nitric acid (HNO_3_, 65%) were purchased from Sinopharm Chemical Reagent Co., Ltd. (Shanghai, China). All measurements were conducted in triplicate.

#### 2.2.7. Statistical Analysis

Pearson correlations among fluoride species during the processes from PG to backfill and from PR to backfill were analyzed using SPSS 27.0 statistical software (IBM SPSS Institute Inc., Armonk, NY, USA, 2020). Correlation coefficients (r) were obtained based on *n* = 5 observations for each correlation. The significance level for all statistical tests was set at 0.05 (*p*  <  0.05) using Duncan’s significant difference test [49].

## 3. Results and Analysis

### 3.1. Total Fluorine Distribution in PR, PG and CPB

**Figure 6:**
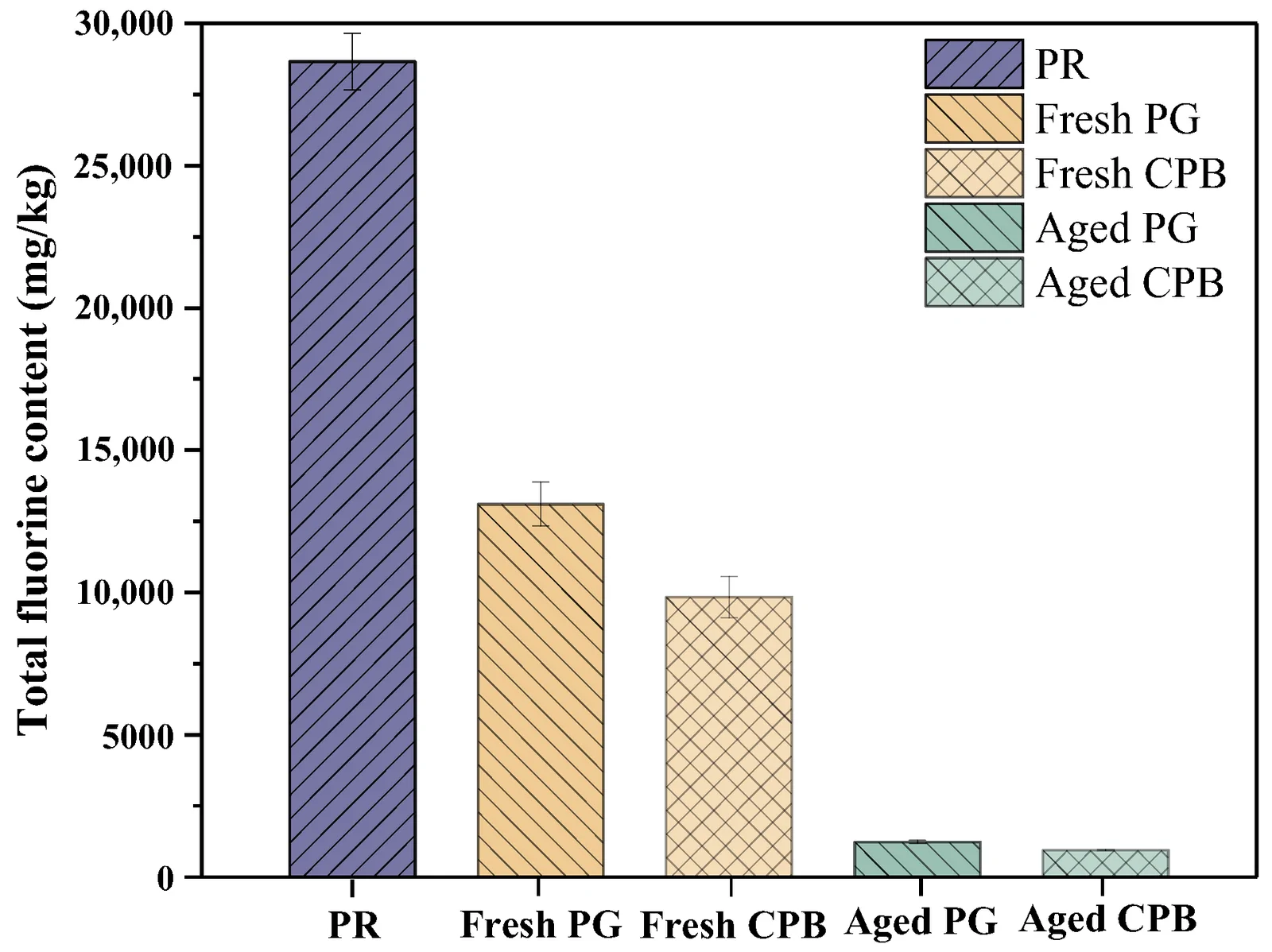
Total fluorine content in PR, PG, and PG-based CPB.

A systematic quantification of TF in PR, PG, and PG-based CPB was performed to elucidate fluorine transformation pathways during cemented backfilling. As shown in Figure 6, TF exhibited a progressive decline from PR to PG and further to CPB.

A high TF of 28,656 mg/kg (2.87 wt%) was measured for the PR samples, which was consistent with the report that PR used for wet-process production typically containing 2–4 wt% fluorine [7]. The elevated fluorine in PR is primarily attributable to fluorapatite (Ca_5_(PO_4_)_3_F), the dominant fluorine-bearing mineral in most sedimentary phosphate deposits. TFs in fresh and aged PG were 13,099 mg/kg and 1232 mg/kg, respectively, representing decreases of 54.29% and 95.70% relative to PR. The substantial loss from PR to fresh PG is primarily attributed to the volatilization of approximately 10–15% of fluorine as gaseous species (e.g., HF, SiF_4_) during the high-acid production process [14]. Subsequently, freshly produced PG is strongly acidic (pH 1–2); therefore, it is subjected to extensive water washing prior to storage, which mitigates acidity and reduces the load of Ws-F [50,51]. This washing process removes a substantial portion of Ws-F from PG due to water–solid interactions, further reducing the TF content in PG. Moreover, the over tenfold difference in TF between fresh and aged PG underscores the significant impact of weathering and storage conditions on PG composition. This ongoing reduction can be attributed to the gradual leaching of Ws-F and the potential formation of volatile fluorides during prolonged atmospheric exposure. Atmospheric processes, such as precipitation and humidity fluctuations, can accelerate this leaching, resulting in a continuous decrease in fluorine content over time [52].

When PG was cemented into the backfill, a further decrease in TF content was observed. TF in CPB prepared with fresh PG was 9830 mg/kg, whereas that of aged PG-based CPB was markedly lower at 936 mg/kg. This nearly tenfold differential is indicative of a pronounced effect of long-term aging on fluorine transformation. Using the modified ETF mentioned in Section 2.2.4, fluorine retention during cementation was high, with values of 94.75% for fresh PG and 93.59% for aged PG. Such high transfer efficiencies indicate that the cemented backfilling process is effective in solidifying/stabilizing most fluoride in PG. However, a minor loss of TF still occurs during the backfill preparation, with losses amounting to 3269 mg/kg for fresh PG and 296 mg/kg for aged PG. Several mechanisms may contribute to this loss. The abrupt pH rise that occurs upon adding alkaline binders during mixing can induce fluoride transformations, generating gaseous fluorides that facilitate volatilization and pose atmospheric risks [34]. Incomplete immobilization during cementation allows unfixed fluorides to escape with bleeding water during dewatering [53]. Early ingress of groundwater during curing can leach fluoride that is not yet bound by C–S–H and AFt/AFm. These pathways may together contribute to the observed TF losses. The pore structure of the cemented backfill is also a primary factor controlling the leaching of fluoride. A finer pore network enhances fluoride immobilization by reducing effective diffusivity and providing abundant adsorption sites [54,55,56].

### 3.2. Fluoride Species in the PR, PG and CPB

**Figure 7:**
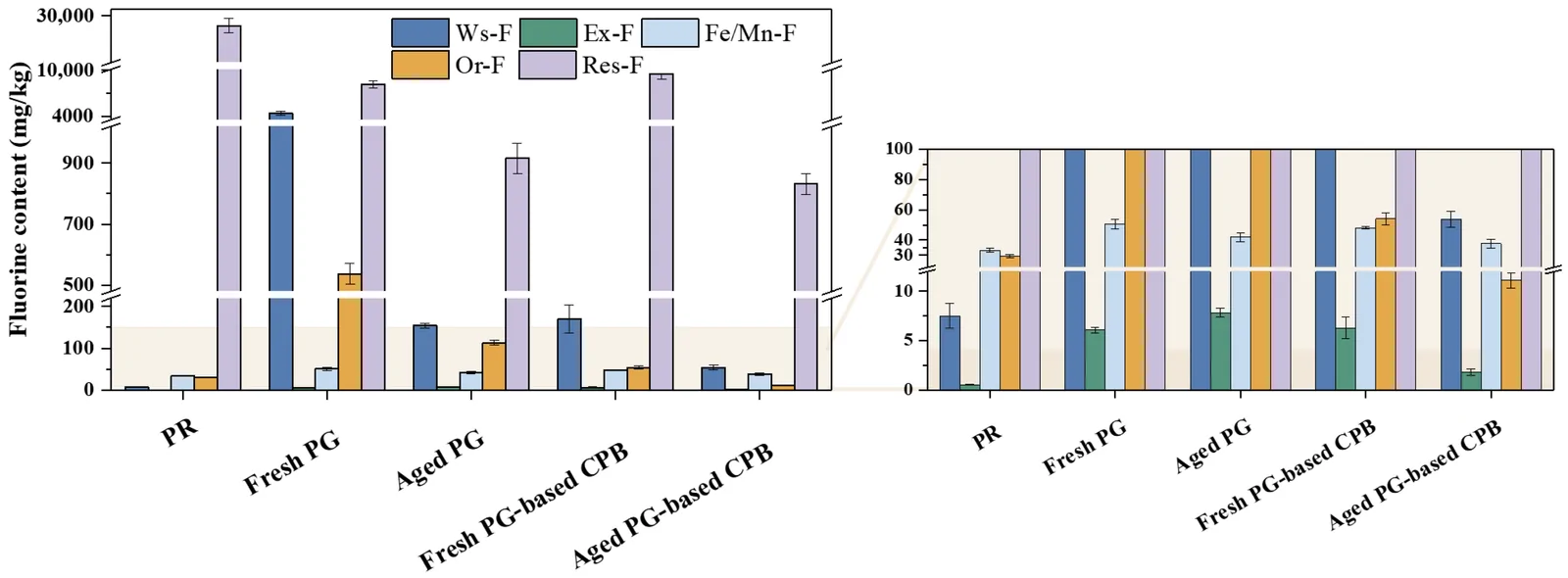
Measured contents of different species of fluorine in PR, PG and PG-based CPB.

To obtain a comprehensive understanding of fluorine behavior and its potential environmental impacts, a modified Tessier five-step sequential extraction procedure was applied to PR, PG, and PG-based CPB, as depicted in Figure 7. This procedure differentiated fluorine into five distinct fractions: Ws-F, Ex-F, Fe/Mn-F, Or-F, and Res-F. The contents and proportions of these fractions relative to TF are listed in Table 1.

In PR, fluorine fractions were ordered as Res-F > Fe/Mn-F > Or-F > Ws-F > Ex-F, with Res-F measured at 28,585 mg/kg, accounting for 99.75% of TF. This dominance was attributed to fluorapatite (Ca_5_(PO_4_)_3_F) serving as the principal and geochemically stable host phase in PR. Under circumneutral conditions, release from fluorapatite is thermodynamically constrained by its low solubility and by Ca^2+^/PO_4_^3−^ buffering, resulting in an overwhelming residual amount of fluorine [57]. The second most abundant fraction, Fe/Mn-F, was 33 mg/kg (0.12%), while other fractions were negligible. Accordingly, fluoride release under natural conditions is expected to be negligible, implying a low pollution potential.

In PG, marked changes in fluorine speciation were observed relative to PR. Res-F remained the dominant fraction, but declined to 62.24% in fresh PG and to 74.23% in aged PG. This shift was accompanied by a marked increase in mobile fluorine, particularly Ws-F and Or-F. Specifically, Ws-F accounted for 33.22% and Or-F for 4.11% in fresh PG, while in aged PG, these contents were 12.53% and 9.81%, respectively. These initial characteristics in fresh PG originate from the production process. In wet-process phosphoric acid production, fluorapatite is decomposed by strong acids, releasing HF, SiF_4_, and soluble fluoro-complexes, while process reagents and residual organics introduce ligands for fluorine complexation. Consequently, fresh PG contained a substantial amount of readily mobilizable fluorine, primarily as Ws-F (33.22%), along with some Or-F (4.11%). A comparison between fresh and aged PG shows pronounced declines in content, with Ws-F decreasing from 4350 mg/kg to 154 mg/kg and Or-F from 537 mg/kg to 113 mg/kg. This result suggests that Ws-F and Or-F are highly mobile and readily leached by rainfall, and the high bioavailability, together with rapid aqueous transport, pose a direct environmental risk [58]. Nevertheless, the persistence of Ws-F and Or-F in aged PG indicates that risk attenuation is incomplete, meaning that overall mobility is reduced by aging, but remaining fluoride in PG may still be released under changing environmental conditions. For Fe/Mn-F and Ex-F, contents in both fresh and aged PG are slightly higher than in PR, which is plausibly related to the redistribution, association, and adsorption of P, Fe, and Mn under secondary conditions following strong-acid dissolution of primary minerals during wet-process production [59].

In CPB, a further shift toward immobile forms was observed, markedly reducing the mobile fractions present in PG. Res-F accounted for 97.17% in CPB prepared with fresh PG and 88.83% in that prepared with aged PG. Correspondingly, the proportions of mobile species were drastically reduced. Ws-F was reduced to 1.71% and 5.76% in the respective CPBs, while Or-F also saw a significant decline to 0.55% and 1.19%. Ex-F and Fe/Mn-F remained minor in both CPB types. These results directly demonstrate a pronounced shift from mobile fluorine fractions to the residual fraction after cementation. Although the specific fluorine-bearing immobilization products were not directly characterized in this study, this transformation may be associated with fluoride retention by cement hydration products and physical encapsulation within the hardened matrix. Previous mineralogical and microstructural studies of PG-based CPB have suggested that C–S–H and AFt/AFm phases may contribute to fluoride retention under cementitious conditions [60]. Fluoride incorporation into stable Ca-bearing fluorine phases during curing has also been reported in related cementitious PG systems [33]. For the Fe/Mn-F fraction, although it was proportionally higher in aged-PG CPB, the absolute Fe/Mn-F content was 38 mg/kg in aged-PG CPB, which was lower than the 48 mg/kg measured in fresh-PG CPB, as presented in Table 1. This finding indicates that the higher proportion likely reflects the lower TF content of aged PG rather than an actual enrichment of Fe/Mn-F. Overall, cementation substantially reduced the relative abundance of mobile fluorine fractions, indicating a reduced potential for fluoride release by leaching.

### 3.3. Fluorine Transformation from PG to CPB

#### 3.3.1. Transformation Characteristics of Fluorine from PG to CPB

To clarify the fluorine transformation during cemented backfilling, species-resolved fluorine content in PG and PG-based CPB was quantified according to Equation (4), focusing on the mobile fractions (Ws-F, Ex-F, Fe/Mn-F, and Or-F) and the immobile fraction (Res-F).

**Figure 8:**
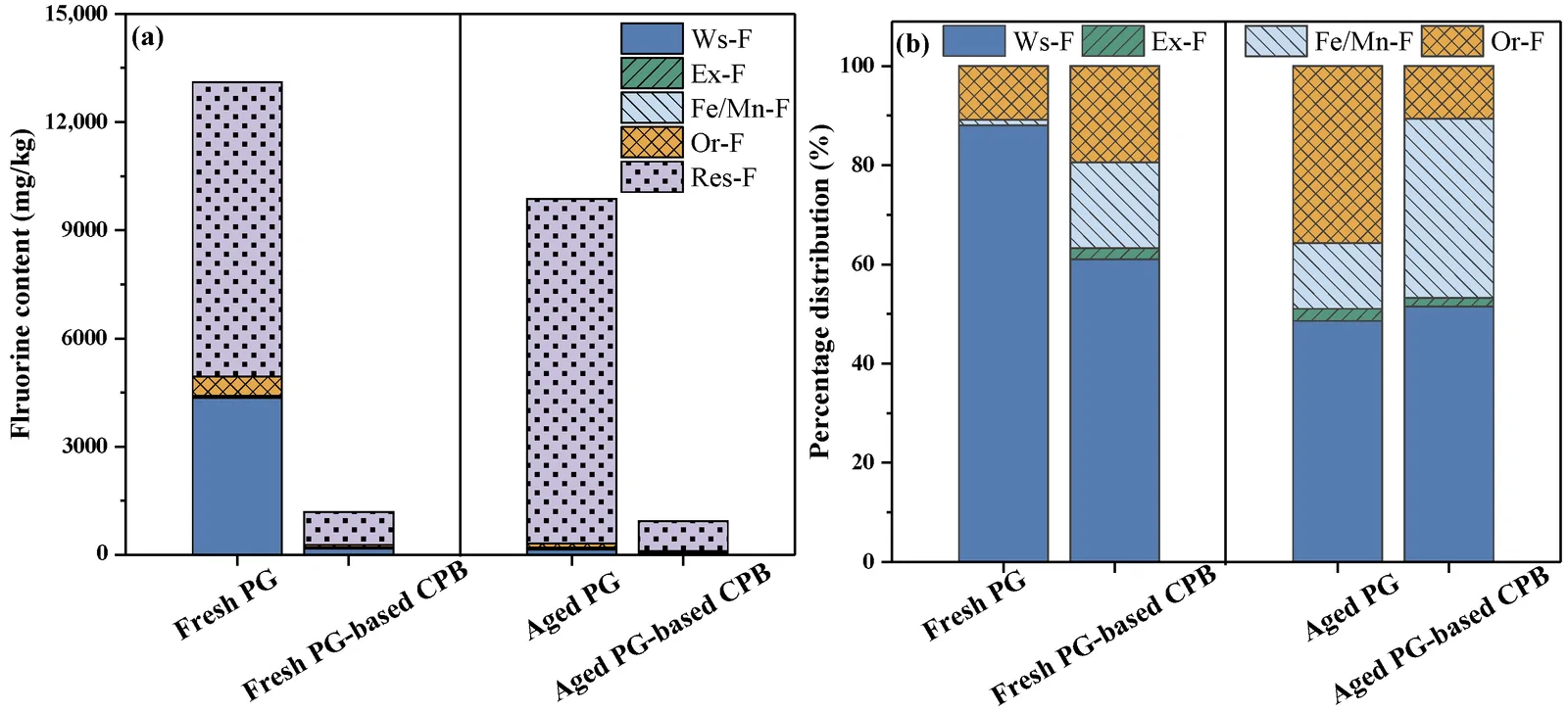
Content and percentage distribution of fluorine species in PG and CPB.

As shown in Figure 8a, the mobile fluorine contents in fresh and aged PG were 4944 mg/kg and 317 mg/kg, respectively, and Res-F was approximately 1.65 and 2.89 times the corresponding mobile fractions. These results indicate that immobile fluorine dominates TF in PG, while the mobile fraction remains substantial (37.75% in fresh PG and 25.73% in aged PG) and controls the potential environmental risk. To further illustrate the fluorine transformation, the percentage distribution of different fluorine species within the mobile fraction is shown in Figure 8b. Within the mobile fraction of PG, Ws-F predominated, with Or-F and Fe/Mn-F also contributing non-negligibly. Specifically, in fresh PG, Ws-F, Or-F, Fe/Mn-F, and Ex-F accounted for 87.99%, 10.86%, 1.03%, and 0.12%, respectively, whereas in aged PG, they accounted for 48.58%, 35.65%, 13.25%, and 2.47%, respectively.

When PG was cemented into the backfill, a marked decrease in the mobile fluorine fraction was observed. The content of mobile fluorine in the fresh and aged PG-based CPB decreased to 278 mg/kg and 104 mg/kg, respectively. Concomitantly, the proportion of Res-F increased, and the immobile-to-mobile ratios rose to about 34 and eight, respectively. These trends, aside from minor fluorine loss during backfill preparation, indicate efficient conversion of mobile fluorine into the immobile fraction through adsorption and complexation on C–S–H and AFt/AFm, physical encapsulation within a refined pore network, and precipitation or re-incorporation into sparingly soluble Ca-F phases such as CaF_2_ and fluorapatite-like structures, followed by progressive lattice fixation during curing. Further analysis revealed that reductions were primarily dominated by Ws-F and Or-F, which together accounted for approximately 98.88% of the total decrease in the mobile fraction in fresh PG and 91.38% in aged PG, suggesting that immobilization is primarily driven by these two species. The substantial reduction in mobile fluorine fractions observed in the present CPB systems is consistent with the fluoride immobilization behavior previously reported for PG-based CPB [33]. This comparison further supports the use of CPB as a risk-mitigation strategy for PG containing a high proportion of mobile fluorine.

#### 3.3.2. Correlation Between Different Fluorine Species in PG and CPB

Following cementation of PG into the backfill, pronounced changes in the contents of different fluorine species were observed, indicating that transformations might have occurred between various species. Correlation coefficients between different fluorine species were calculated to quantify the transformation, and the results are shown in Table 2 and Table 3.

As shown in Table 2, a significant correlation (*r* = 0.995) was observed between Ws-F and Or-F in fresh PG and the resulting backfill, suggesting a strong potential for interconversion between these two species during the cemented backfill process. Both belong to the mobile fraction and are particularly susceptible to changes driven by cementation reactions and sorption processes on hydration products. The correlations between Ex-F and Fe/Mn-F and other fluorine species in Table 2 were weaker, indicating that Ex-F and Fe/Mn-F remained comparatively stable. In aged PG, similar trends were identified in the correlation coefficients presented in Table 3. Ws-F exhibited a significant correlation with both Ex-F and Or-F, and Ex-F also showed a strong correlation with Or-F. The strong correlations among Ws-F, Ex-F, and Or-F indicate a dynamic redistribution among aqueous, exchangeable, and ligand-bound fractions that is sensitive to ionic strength and Ca^2+^ activity and to the availability of sorption sites, followed by capture by C–S–H and AFt/AFm and precipitation of low-solubility Ca–F phases. The weak correlation observed between Fe/Mn-F and Res-F with other species indicates that both fractions are relatively stable. Res-F is structurally bound and controlled mainly by mineral saturation constraints, whereas Fe/Mn-F functions as a secondary retention pool that changes little across the transition from the acidic PG environment to the alkaline CPB environment. It is noteworthy that differences between the fresh and aged systems likely arise from distinct fluorine contents and speciation, which create different chemical environments for cementation reactions and for sorption capacity.

### 3.4. Net Change and Redistribution of Fluorine from PR to CPB

#### 3.4.1. Transformation Characteristics of Fluorine from PR to CPB

**Figure 9:**
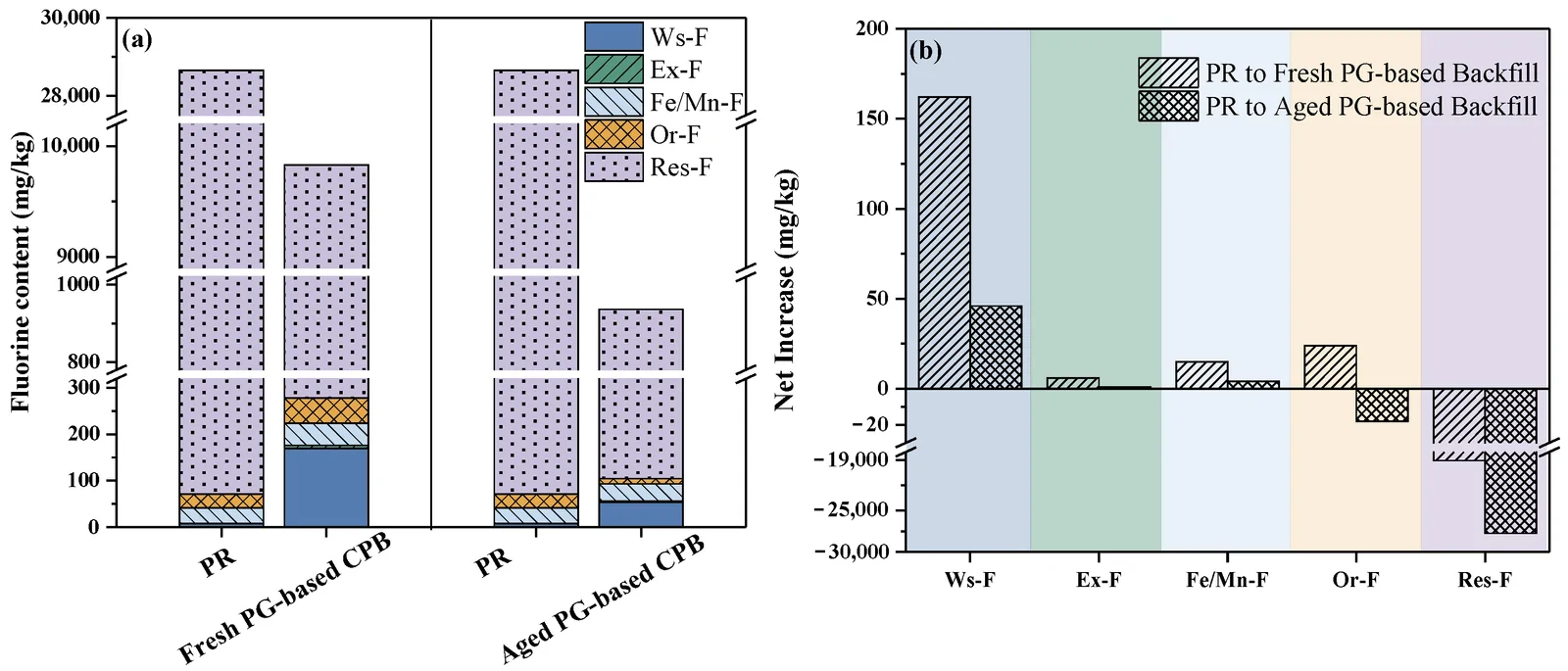
Content (**a**) and net change (**b**) of fluorine species from PR to CPB.

While the above analysis detailed the sequential transformation of fluorine speciation from PR to PG and then to CPB, a net change analysis from the PR to the CPB provides a holistic assessment of the efficacy and reveals non-intuitive dynamics in the cementation process. The net changes in fluorine species from PR to CPB, calculated as the difference in content between CPB and PR, are summarized in Figure 9. A critical finding is that contrary to the substantial overall decrease in TF, the content of mobile fluorine exhibited a net increase of 207 mg/kg in fresh PG-based CPB and 33 mg/kg in aged PG-based CPB, as seen in Figure 9b. This increase was driven predominantly by the rise in Ws-F, which increased by 162 mg/kg and 46 mg/kg in the fresh and aged PG-based CPB, respectively. Ex-F and Fe/Mn-F fractions also showed minor increments in the range of 1 to 15 mg/kg. Concomitantly, Res-F exhibits a large net decrease by 19,033 mg/kg and 27,753 mg/kg in the fresh and aged systems, indicating redistribution from lattice-fixed structures. This net mobilization within an overall immobilizing process underscores that cementation, while highly effective in reducing mobile fluorine inherited from PG, does not fully reverse the activation that occurs during the wet-process conversion of PR to PG. A small but persistent reservoir of newly mobile fluorine remains in the CPB matrix. In the final CPB, mobile fluorine accounts for 2.8% of TF in the fresh system and 11.2% in the aged system, with Ws-F alone at 1.7% and 5.8%, respectively. This residual mobile fraction is susceptible to leaching under hydrologically active conditions and underscores the need to optimize formulations and curing to suppress secondary mobilization from reactive phases.

#### 3.4.2. Correlation Between Different Fluorine Species in PR and CPB

To further elucidate the complex redistribution suggested by the net change analysis, correlation coefficients between fluorine species across the PR to CPB transition were examined, as shown in Table 4 and Table 5. The patterns are indicative that transformation is not a simple one-way fixation and that multifaceted interconversions among species are involved. Strong negative correlations between Res-F and all mobile fractions with r less than −0.89 are consistent with depletion of the residual fraction coupled with activation of mobile fractions along the production and cementation process. In the fresh system, strong covariation with mixed signs was observed among mobile species, with most pairs showing positive correlations, and Ws-F versus Ex-F being strongly negative, whereas uniformly strong positive associations were observed in the aged system. These features are consistent with active redistribution within the mobilized fraction through ligand exchange, adsorption–desorption, and precipitation–dissolution under alkaline and Ca-rich conditions during cementation. Differences in correlation strength and sign between the fresh and aged PG-based CPB indicate that redistribution pathways in CPB are preconditioned by initial PG speciation and its aging. Overall, the correlations support the findings of net change. First, substantial depletion of the Res-F occurs concurrently with the overall TF decrease throughout processing. Second, a chemistry-dependent reshuffling persists within the mobile fraction, part of which remains in the final CPB and governs residual leaching risk.

## 4. Discussion

### 4.1. Dynamics of Fluorine Speciation and Environmental Risk

TF exhibited a marked reduction across processing stages, decreasing from PR to PG and further decreasing in CPB. This continuous reduction is likely driven by coupled fluoride-release pathways along the production chain, including dissolution and washout during PG storage, aging and partial loss during CPB preparation. Because environmental risk is governed by speciation, changes within the mobile fraction (Ws-F, Ex-F, Fe/Mn-F, Or-F) relative to the immobile fraction (Res-F) are decisive. Compared with PR, the increase in mobile fluorine fractions observed in CPB, particularly the notable rise in Ws-F, indicates a residual mobile fraction that may be susceptible to leaching under subsurface conditions. This result is consistent with previous studies showing that CPB can substantially reduce soluble fluoride release from PG, but may not completely eliminate fluoride leaching [19,31]. Therefore, understanding the interrelationships between different fluoride species becomes particularly important. The strong correlation between Ws-F and Or-F likely reflects ligand-exchange and complexation equilibria that are sensitive to ionic strength, Ca^2+^ activity and the availability of sorption sites on C-S-H and AFt/AFm, such that changes in one fraction can propagate to the other. Res-F exhibits distinct behavior across stages. The net mass decreases when tracking from PR to CPB because of earlier losses during the PG stage, whereas the proportion increases during the PG to CPB step as mobile fluorine is immobilized on hydration products. These findings emphasize the need to consider dynamic interconversion among fluoride species when controlling fluorine pollution. Differences in correlation patterns between fresh and aged PG-based CPB likely arise from aging-induced changes in initial inventories and surface chemistry, including recrystallisation, reduced ionic strength and modified sorption capacities, which alter the relative responsiveness of Ws-F, Ex-F and Or-F. Such differences may also explain variations in fluoride immobilization performance reported among previous studies, since PG source, storage history, and initial fluoride speciation can differ substantially [19,52]. Binder composition and curing conditions may further affect fluoride retention in PG-based CPB [33,53]. Consistent variation in immobilization performance and in the proportions of fluoride species during transformation indicates that fluorine release is time-dependent. Storage aging modifies the reaction environment for subsequent cementation reactions and for sorption and precipitation pathways. A mechanistic understanding that couples surface complexation, precipitation–dissolution and pore-scale transport can support predictive release models and inform backfill mix design and processing windows.

### 4.2. Fluorine Immobilization and the Role of the Cemented Backfill Technique

The cemented backfill technique shows substantial potential for mitigating fluorine-related environmental risks. A high ETF of fluorine during the cementation process indicates that a large portion of fluorine from PG can be retained through solidification and stabilization. The finding is in agreement with earlier studies reporting marked reductions in soluble fluoride concentrations after PG is incorporated into CPB [56,60]. The observed TF loss may be related to the removal of dissolved fluorine with bleeding water or other material losses during backfill preparation. However, the specific loss pathways were not determined in this study. Therefore, targeted control measures, particularly the management of bleeding water, may help reduce fluorine loss during backfill preparation. Further optimization of early hydration, including the formation of C–S–H and AFt/AFm phases and the availability of Ca and Al, may also enhance fluoride retention before setting. The shift from mobile fluorine fractions to the residual fraction may be associated with fluoride retention by cement hydration products, physical encapsulation within the hardened matrix, and/or the formation or stabilization of sparingly soluble Ca-bearing fluorine phases. These mechanisms have been proposed in previous studies of cementitious PG systems. However, the specific fluorine-bearing phases were not directly identified in the present study. Further investigations using mineralogical, microstructural, and spectroscopic techniques are therefore needed to clarify the mechanisms of fluoride retention. Based on the present findings, potential directions for future optimization include adjusting binder composition to increase Ca and Al availability, optimizing the water-to-solids ratio, and pre-treating PG to reduce labile fluoride prior to cementation. The effects of curing humidity, temperature, and sulfate activity on fluorine immobilization and long-term leaching behavior should be systematically investigated.

In conclusion, cemented backfilling can substantially mitigate fluorine-related environmental risks when residual mobile fractions are minimized through informed mix design and controlled curing. A mechanistic understanding of fluorine speciation, sorption, and precipitation pathways enables tighter control over its release, supporting more sustainable phosphate mining and processing.

## 5. Conclusions

This study provides a systematic, species-resolved investigation of fluorine transformation and environmental implications along the phosphate production and waste stabilization chain, from PR to PG and finally to PG-based CPB. The principal conclusions are as follows:

(1) TF decreased from PR to PG and further to CPB. However, net change analysis revealed that mobile fluorine, particularly Ws-F, was higher in CPB than in PR, indicating that fluorine mobilization during wet-process conversion was not fully reversed by cementation.
(2) Fluorine speciation changed substantially along the PR–PG–CPB process chain. Fluorine in PR was predominantly present as Res-F, whereas PG contained higher proportions of mobile fractions, mainly Ws-F and Or-F. After cementation, Res-F became the dominant fraction in CPB, accounting for 88.83–97.17% of TF.
(3) Cementation achieved a high fluorine ETF exceeding 93%, indicating that most fluorine in PG was retained in CPB. Relative to PG, cementation generally decreased mobile fluorine fractions, indicating reduced potential fluorine mobility.
(4) Ws-F remained in CPB, accounting for 2.8–11.2% of TF, indicating that a potentially mobile fluorine fraction persisted after cementation. Differences between CPB prepared from fresh and aged PG further demonstrate that PG storage history influences fluorine fractionation and retention.

## Figures and Tables

**Table 1:** Content and proportion of fluorine species in PR, PG and PG-based CPB.

Sample	Ws-F		Ex-F		Fe/Mn-F		Or-F		Res-F	
	C (mg/kg)	P (%)	C (mg/kg)	P (%)	C (mg/kg)	P (%)	C (mg/kg)	P (%)	C (mg/kg)	P (%)
PR	7.51	0.03	0.51	0.002	33	0.12	30	0.10	28,585	99.75
Fresh PG	4350	33.22	6.07	0.05	51	0.39	537	4.11	8154	62.24
Aged PG	154	12.53	7.83	0.64	42	3.42	113	9.81	915	74.23
Fresh PG-based CPB	170	1.71	6.29	0.06	48	0.49	54	0.55	9552	97.17
Aged PG-based CPB	54	5.76	1.81	0.19	38	4.03	11	1.19	832	88.83

C: content; P: proportion.

**Table 2:** Correlation coefficients between fluorine species in fresh PG and the fresh PG-based CPB.

Species	Ws-F	Ex-F	Fe/Mn-F	Or-F	Res-F
Ws-F	1				
Ex-F	−0.133	1			
Fe/Mn-F	0.473	−0.353	1		
Or-F	0.995 *	−0.103	0.392	1	
Res-F	−0.713	0.070	−0.050	−0.726	1

* *p*  <  0.01.

**Table 3:** Correlation coefficients between fluorine species in aged PG and the aged PG-based CPB.

Species	Ws-F	Ex-F	Fe/Mn-F	Or-F	Res-F
Ws-F	1				
Ex-F	0.973 *	1			
Fe/Mn-F	0.508	0.634	1		
Or-F	0.993 *	0.990 *	0.543	1	
Res-F	0.658	0.737	0.260	0.716	1

* *p*  <  0.01.

**Table 4:** Correlation coefficients between fluorine species in PR and the fresh PG-based CPB.

Species	Ws-F	Ex-F	Fe/Mn-F	Or-F	Res-F
Ws-F	1				
Ex-F	−0.994 *	1			
Fe/Mn-F	0.973 *	0.944 *	1		
Or-F	0.996 *	0.999 *	0.949 *		
Res-F	−0.991 *	−0.994 *	−0.957 *	−0.990 *	1

* *p*  <  0.01.

**Table 5:** Correlation coefficients between fluorine species in PR and the aged PG-based CPB.

Species	Ws-F	Ex-F	Fe/Mn-F	Or-F	Res-F
Ws-F	1				
Ex-F	0.991 *	1			
Fe/Mn-F	0.870 *	0.893 *	1		
Or-F	0.996 *	0.993 *	0.845 *		
Res-F	−0.997 *	−0.995 *	−0.892 *	−0.994 *	1

* *p*  <  0.01.

## Data Availability

The original contributions presented in this study are included in the article. Further inquiries can be directed to the corresponding author.

## Abbreviations

PG	Phosphogypsum
PR	Phosphate rock
CPB	Cemented paste backfill
Ws-F	Water-soluble fluorine
Ex-F	Exchangeable fluorine
Fe/Mn-F	Iron-manganese oxide fluorine
Or-F	Organically bound fluorine
Res-F	Residue fluorine
